# Editorial: Gut microbiota and gut-associated metabolites in bone health

**DOI:** 10.3389/fendo.2023.1232050

**Published:** 2023-07-20

**Authors:** Rupesh K. Srivastava, Niharika Arora Duggal, Narayanan Parameswaran

**Affiliations:** ^1^ Translational Immunology, Osteoimmunology and Immunoporosis Lab, Department of Biotechnology, All India Institute of Medical Sciences (AIIMS), New Delhi, India; ^2^ Medical Research Council (MRC)-Arthritis Research UK Centre for Musculoskeletal Ageing Research, Institute of Inflammation and Ageing, University of Birmingham, Birmingham, United Kingdom; ^3^ Department of Physiology, Michigan State University, East Lansing, MI, United States

**Keywords:** gut microbiota, gut-associated metabolites, inflammation, osteoporosis, osteoarthritis, rheumatoid arthritis

## Introduction

The term microbiota refers to all the microbes (including bacteria, archaea, viruses, and eukaryotes) that exist in a particular niche in/on the host. Microbes cover all the host mucosal surfaces with the majority of them residing within the gastrointestinal tract (gut microbiota). The human gut microbiota comprises of ~100 trillion microbes (500-1000 species) which encode for more than 3.3 million genes. These microorganisms have co-evolved with humans and improve human health by regulating several biological processes. Establishing and sustaining beneficial exchanges between the host and its accompanying microbiota are the key requirements for the maintenance of the healthy life of the host. Gut microbiota regulates host physiology and development, influences host metabolism, and modulates the host immune system (1, 2). Studies over the last decade have revealed a novel nexus between gut microbiota and bone homeostasis (3–5). Importantly, recent investigations have provided further evidence that gut microbiota and its associated metabolites (GAMs) modulate bone metabolism and several metabolic bone diseases such as osteoporosis, osteoarthritis, and rheumatoid arthritis (RA). The current Research Topic “*Gut microbiota and Gut-associated metabolites in bone health*” highlights the importance of the latest research in this emerging and interesting field of Osteomicrobiology. This Research Topic contains 4 contributions with authors from China and India. The compilation of these recent reviews and research articles will solidify our knowledge regarding the role of GM and GAMs in the maintenance of bone homeostasis. This Research Topic primarily consists of the following specific topics.

## Gut microbiota and bone health

Gut microbiota influences the homeostasis of several tissues including bone. Numerous studies in the past decade have evidenced the significance of gut microbiota in the regulation of bone health. Guan et al. in their review focused on several mechanisms underlying gut microbiota-mediated regulation of bone health. One such mechanism is gut microbiota facilitating nutrient absorption as well as help to maintain intestinal barrier integrity which further enhances bone mineral density (BMD). In addition, microbiota regulates the immune system which has a significant role in maintaining skeletal homeostasis. Tu et al. also reviewed another novel mechanism referred to as the entero-endocrine-osseous axis to summarize the interaction of gut microbiota with the endocrine system to promote bone health. Gut microbiota influences several bone metabolism-regulating hormones to maintain skeletal homeostasis. For instance, the importance of estrogen in preserving bone health is well known. Estrogen reduces bone resorption by maintaining systemic and bone marrow T cells homeostasis in addition to directly modulating the formation of osteoblasts and osteoclasts. The gut microbiota controls the metabolism of estrogen and raises its circulating levels. Another hormone testosterone can both reduce the apoptosis of osteoblasts and enhance the proliferation of osteoblast precursors. Probiotics administration and fecal microbiota transplantation can control testosterone levels while concurrently changing bone structure. Microbiota also influences other key bone-regulating hormones such as insulin-like growth factor-1, parathyroid hormone, serotonin, and gastrointestinal hormones. Taken together, it is clear that gut microbiota plays a crucial role in maintaining bone metabolism and it does so by interacting with several physiological systems in the body.

There are several methods to dissect the effect of gut microbiota on bone health. Depletion of gut microbiota with a selective mixture of antibiotics or use of germ-free mice are some of the most common strategies to evaluate the effect of gut microbiota on bone health in various disease conditions. Guan et al. reviewed the role of depletion of gut microbiota in bone pathologies such as osteoporosis and osteoarthritis. On the other hand, other studies pointed out the beneficial role of gut microbiota in maintaining bone health. Taken together the role of gut microbiota is very complex and conflicting in the perspective of bone health and thus further research is still warranted and required to dissect the complete nexus between gut microbiota and bone health under both physiological and pathological conditions.

## Association of gut microbial composition and bone health

Recent studies have highlighted that gut microbial composition significantly impacts bone health. Guo et al. reported that perturbation of gut microbiota composition with antibiotic treatment resulted in decreased BMD. However, probiotic treatment with *Lactobacillus casei* fermented milk reversed the effect of antibiotics and promoted fracture healing in osteoporotic mice highlighting the important role of gut microbiota composition in bone health. The association of gut microbiota composition with several bone ailments is being actively investigated by several groups. Dagar et al. reviewed the role of dysbiosis in RA. The primary bacterial family in the gut most closely connected with RA is *Prevotellaceae*. *Prevotella copri* overgrowth was seen in several investigations in RA patients’ fecal samples. One major lacuna that Dagar et al. highlight in their review is that the majority of current studies in the field of RA have been devoted to describing the role of gut bacteria. Research on gut fungal and virome components in RA is a relatively new and developing subject. However, alterations in fungal and viral components likely have a significant effect on the development of RA. Dagar et al. filled this gap and documented the role of various viral and fungal components in the pathogenesis of RA. Altogether the composition of complete gut microbiota (i.e., bacterial, fungal, and viral) is an important factor in the regulation of bone homeostasis.

## Treatment modalities for gut microbiota associated bone pathologies

As dysbiosis is associated with several bone pathologies, modulation of gut microbiota composition could be an effective strategy for the prevention, management and perhaps the treatment of these bone pathologies. Gut microbiome-modifying medications suggest that altering the gut microbiome may be a promising therapeutic or adjunct approach for the treatment of RA. Interventions such as administration of probiotics, prebiotics, and fecal microbiota transplantation are the preferred methods for restoring the dysregulated gut microbiota. Numerous studies have highlighted the role of these interventions as a therapeutic strategy for various inflammatory bone conditions. However recently the GAMs are receiving significant attention as a treatment modality for gut microbiota-associated pathologies including bone disorders. GAMs that are considered the most prevalent are short-chain fatty acids (SCFAs) and bile acids (BAs). In recent years, a number of studies have underscored the role of SCFAs in skeletal homeostasis. It has been shown that SCFAs block histone deacetylases (HDACs) and activate free fatty acid receptors (FFARs) to prevent the development of osteoclasts. In addition to their direct impact on bone cells, SCFAs promote the differentiation of regulatory T cells while inhibiting osteoclastogenic Th17 cells. The crucial involvement of BAs in bone remodeling has been demonstrated by the positive connection between serum levels of total BAs and BMDs in postmenopausal women. BAs enhance osteogenic differentiation while inhibiting osteoclastogenesis (Tu et al.). Taken together, interventions targeting the gut microbiota are effective strategies for bone loss, and gut microbiota modifying therapies, especially the gut metabolites can be examined for their effectiveness at the clinical level in the near future.

## Conclusion

Gut microbiota has the potential of regulating bone homeostasis and dysbiosis of gut microbial composition can lead to several bone pathologies. Modifying gut microbial composition can thus be a promising or adjunct strategy for the treatment and management of various skeletal disorders. However, large-scale clinical trials are required to clearly define their efficacy in humans.

## Author contributions

All authors listed have made a substantial, direct, and intellectual contribution to the work and approved it for publication.
